# Supplementary material for: Analysis of PPARγ Signaling Activity in Psoriasis
Source: Int J Mol Sci. 2021 Aug 10;22(16):8603. doi: 10.3390/ijms22168603 (PMC8395241; doi:10.3390/ijms22168603)
Supplement: Supplementary file 1 [file ijms-22-08603-s001.zip › Supplemental materials_Analysis of PPARg signaling activity in psoriasis/Pathway models/Models images and html files/PPARG expressed in cells assotiated with psoriasis/211433.html]

lymphatic system


# Organ lymphatic system

|  |  |
| --- | --- |
| URN | urn:agi-ncimorgan:C0024235 |
| Total Entities | 7 |
| Connectivity | 5837 |
| Name | lymphatic system |

---

|  |  |
| --- | --- |
| ChildConcepts | lymphatic vessels |
|  | lymphoid tissue |
|  | lymphoid organ |
|  | lymph |
|  | cisterna chyli |
|  | lymphoid cell |
|  | lymphatic stoma |

---

|  |  |
| --- | --- |
| Pathway | PPARG expressed in cells assotiated with psoriasis |
|  | Neighbors of lymphatic system |

---

|  |  |
| --- | --- |
| MedScan ID | 8800798 |

---

|  |  |
| --- | --- |
| Alias | lymphatic |
|  | lymphatic tract |
|  | lymphoids |
|  | lymphatic systems |
|  | lymphatics |
|  | lymphoid systems |
|  | lymphoid |
|  | lymphatic trunk |
|  | lymphatic tracts |
|  | lymphatic trunks |
|  | systema lymphaticum |
|  | lymph system |
|  | lymph systems |
|  | lymphoid system |
|  | lymphatic tree system |
|  | lymphatic system |

---

|  |  |
| --- | --- |
| NCIm ID | C0024235 |

---
